# Charcot–Marie–Tooth mutation in glycyl-tRNA synthetase stalls ribosomes in a pre-accommodation state and activates integrated stress response

**DOI:** 10.1093/nar/gkab730

**Published:** 2021-08-17

**Authors:** Samantha Mendonsa, Nicolai von Kuegelgen, Lucija Bujanic, Marina Chekulaeva

**Affiliations:** Berlin Institute for Medical Systems Biology, Max Delbrück Center for Molecular Medicine in the Helmholtz Association, Berlin, Germany; Free University, Berlin, Germany; Berlin Institute for Medical Systems Biology, Max Delbrück Center for Molecular Medicine in the Helmholtz Association, Berlin, Germany; Free University, Berlin, Germany; Berlin Institute for Medical Systems Biology, Max Delbrück Center for Molecular Medicine in the Helmholtz Association, Berlin, Germany; Berlin Institute for Medical Systems Biology, Max Delbrück Center for Molecular Medicine in the Helmholtz Association, Berlin, Germany

## Abstract

Toxic gain-of-function mutations in aminoacyl-tRNA synthetases cause a degeneration of peripheral motor and sensory axons, known as Charcot–Marie–Tooth (CMT) disease. While these mutations do not disrupt overall aminoacylation activity, they interfere with translation via an unknown mechanism. Here, we dissect the mechanism of function of CMT mutant glycyl-tRNA synthetase (CMT-GARS), using high-resolution ribosome profiling and reporter assays. We find that CMT-GARS mutants deplete the pool of glycyl-tRNA^Gly^ available for translation and inhibit the first stage of elongation, the accommodation of glycyl-tRNA into the ribosomal A-site, which causes ribosomes to pause at glycine codons. Moreover, ribosome pausing activates a secondary repression mechanism at the level of translation initiation, by inducing the phosphorylation of the alpha subunit of eIF2 and the integrated stress response. Thus, CMT-GARS mutant triggers translational repression via two interconnected mechanisms, affecting both elongation and initiation of translation.

## INTRODUCTION

Defects in translational regulation have been identified as common features in multiple neurodegenerative disorders (reviewed in (1)). Yet in some cases the precise mechanisms by which they disrupt translation have to be clarified. For protein synthesis, amino acids are ligated to their cognate tRNAs by aminoacyl-tRNA synthetases (aaRSs), and mutations in six of these enzymes cause a degeneration of peripheral motor and sensory axons, known as Charcot–Marie–Tooth (CMT) disease (reviewed in (2)). A subtype of this disease, CMT type 2D (CMT2D), is caused by dominant mutations in the gene encoding glycyl-tRNA synthetase (*GARS*). Curiously, although overall aminoacylation activity is not disrupted by CMT2D-causing mutations, global translation is inhibited (3–7). This raises the question about the mechanisms of translational repression in this disease.

Translation cycles through three stages: initiation, elongation and termination. Because initiation is the rate-limiting stage of translation, it has been considered the main stage at which translational control occurs (reviewed in (8)). Eukaryotic translational initiation is a multi-step process that requires many proteins, the so called eukaryotic initiation factors (eIFs). It involves the formation of the 43S pre-initiation complex, which consists of the small (40S) ribosomal subunit, ternary complex (the initiator Met-tRNAi and eIF2 in its GTP-bound form, hereafter referred to as eIF2:GTP:Met-tRNAi), and other factors. The 43S complex is recruited to the mRNA 5′-end and scans the mRNA until it finds the initiation AUG codon and can bind the large (60S) ribosomal subunit. Joining of the large (60S) ribosomal subunit completes formation of the 80S ribosome with aminoacylated tRNA in the ribosomal P-site.

Most of the steps of initiation can be regulated. A major regulatory mechanism is triggered by various stress conditions and is called the integrated stress response (ISR, reviewed in (9)). It involves the phosphorylation of the alpha subunit of eIF2 (eIF2a), which reduces the levels of the ternary complex eIF2:GTP:Met-tRNAi. This leads to a downregulation of global translation initiation, to save cellular resources under stress conditions, and the upregulation of specific transcripts, such as Activating transcription factor 4 (ATF4), required to fix stress-related damage (10–12). A number of reports have found that ISR is activated in neurodegenerative diseases, including Alzheimer's and prion disorders (13), amyotrophic lateral sclerosis (14) and cerebellar and retinal degeneration (15).

Recent evidence has shown that the stage of elongation of translation can also be targeted by complex regulatory mechanisms, and that this plays important roles in development and neurologic diseases (reviewed in (16)). Mutation in the ribosome rescue factor GTPBP2, underlying cerebellar and retinal degeneration (17), has been associated with ribosome stalling during elongation (15). Similar effects have been reported for FMRP-linked disorders, Fragile X syndrome and autism (18). Elongation requires two eukaryotic elongation factors (eEFs) and consists of three main steps: (i) the accommodation of the aminoacylated tRNA (aa-tRNA), in complex with eEF1A:GTP, into the A-site of the ribosome, (ii) the formation of the peptide bond, catalyzed by the large ribosomal subunit, during which the growing polypeptide from the P-site is transferred to aa-tRNA in the A-site, (iii) ribosome translocation, catalyzed by eEF2, during which peptidyl-tRNA moves to the P-site and deacylated tRNA is evicted from the P-site. Ribosomes undergo major conformational rearrangements during elongation, and recent works have shown that ribosome profiling can distinguish between two functional states of the ribosome—before and after aa-tRNA binding (19,20).

Here, we dissect the mechanism of translational regulation by CMT2D-causing mutations in GARS (CMT-GARS). Using high-resolution ribosome profiling, we show that CMT-GARS mutant G240R causes ribosomes to stall at glycine codons in open A-sites, due to increased retainment of tRNA^Gly^ on mutant CMT-GARS and a shortage of glycyl-tRNA^Gly^ available for translation. Moreover, ribosome stalling triggers a secondary translational repression mechanism, which involves an increase in the phosphorylation of eIF2α and induction of ISR.

## MATERIALS AND METHODS

### Cell culture, transfections, and luciferase assay

Human HEK293T cells were grown in Dulbecco's modified Eagle's medium with GlutaMAX™ supplement (DMEM + GlutaMAX, GIBCO) with 10% FBS. Transfections were done in 10 cm, 6-well and 96-well plates with polyethylenimine (PEI) using a 1:3 ratio of DNA:PEI. In reporter experiments, HEK293T cells were transfected with 1–2 ng RL or RL-ATF4, 10 ng FL and 10 ng GARS-myc constructs per well of a 96-well plate. Total amount of transfected DNA was topped up to 50 ng per well of 96-well plate with the empty vector. For other formats, the amounts of plasmids were adjusted proportionally. For myc immunoprecipitation, amounts of GARS-expressing plasmids were adjusted to achieve equal expression levels (1.5 μg WT, 3 μg E71G and 7.5 μg G240R and 10 μg ΔETAQ GARS-myc per 10 cm plate), and amount of transfected DNA was topped up to 10 μg with the empty vector. Cells were lysed 24 h post transfection. Luciferase activities were measured with a homemade luciferase reporter assay system as described earlier (21). For puromycylation assay, cells were treated with 2.5 μg puromycin for 30 min before lysis. Where indicated, thapsigargin was added at 50 nM for 30 min before cell lysis and GCN2-IN-1 at 1 μM at the time of transfection.

### Ribosome profiling

Ribosome profiling was performed as earlier described (22), with the following modifications. Monosomes were purified using Microspin S-400 HR columns (GE Healthcare 27-5140-01) and 15–35 nt ribosome-protected fragments were isolated for library generation.

### DNA constructs

Reporter plasmids RL and FL have been described previously (23). ATF4-RL reporter was generated by PCR amplifying *ATF4* 5′UTR (ENSMUST00000109605.5) and cloning between SacI and NheI of RL. To generate GARS-myc-expressing plasmid, blasticidin resistance CDS was PCR amplified and cloned between SbfI and SanDI of piggyBac vector pCyl50-MCS (kind gift of Dr Julien Bethune (24)), to generate piggyBac-Blast. GARS CDS (P41250-2) was PCR amplified from human cDNA and cloned between FseI and AgeI sites of piggyBac-Blast. E71G, G240R and ΔETAQ (245-248) mutations were introduced in GARS CDS by site-directed mutagenesis. To generate 3xflag-NSP1-encoding plasmid, a synthetic 3xflag sequence was cloned between BstXI and SbfI sites of pEBG-sic plasmid (25) to produce pEBG-3xflag. CDS of NSP1 was PCR amplified, using SARS-CoV2 cDNA as a template, and cloned between SbfI and NotI sites of pEBG-3xflag.

### PAGE and northern blotting

For aminoacylation level experiments, total RNA from 293T cells expressing GARS-myc was isolated with Trizol (Thermo), according to the manufacturer's instructions, and resuspended in 1 mM sodium acetate pH 5.0. A portion of each sample was subjected to deacylation by addition of 0.2 M Tris–HCl pH 9.5 and incubation at 37°C for 30 min. 1 μg of total RNA per sample was further analyzed by acid–urea PAGE and northern blotting as described earlier (26). More specifically, the samples were separated on a 40 cm × 40 cm 10% PAAG (AA:MBA = 19:1) prepared in 0.1 M sodium acetate (pH 5.0) and 8 M urea. The gel was run at 120 V for 18 h at 4°C, until the bromophenol blue dye ran out. For analysis of GARS-myc immunoprecipitates, samples were run on 10% TBE–urea PAAG at 200 V for 1 h, and 400 ng of total RNA were loaded as inputs. RNA was then transferred to a Hybond-N + membrane (Amersham) using semi-dry transfer in 1× TBE buffer at 15 V for 1 h. The membrane was rinsed in 5× SSC buffer and RNA was crosslinked to the mebrane using Stratalinker (265 nm) at 120 000 μJ/cm^2^. The membrane was pre-hybridyzed in 6× SSC, 10× Denhardt solution, 0.5% SDS at 42°C for 1 h. Hybridization was done in 6× SSC, 0.1% SDS and 20 pmol of radiolabeled probe at 45°C overnight. The membrane was then washed with 2× SSC three time for 10 min at room temperature and exposed with the phosphorimager screen for 4 h to overnight. The following oligonucleotides were used as probes for northern blotting: TCTACCACTGAACCACCAATGC (tRNA^Gly (GCC)^); CAGCCAGATCGCCCTCACATCC, CAGCCAGATCAGCCGAATCAAC, TCTTCGACCGAGCGCGCAGCTT and CTTGAGAGCTTGTTTGGAGGTT (7SK); TAGGTCAGGGTGGTCACGAG, TGGCGGACTTGAAGAAGTCG, CTTGAAGAAGATGGTGCGCT, TGAACTTGTGGCCGTTTACG (GFP). To prepare the probes, 20 pmol of oligonucleotide (tRNA^Gly^) or oligonucleotide pool (7SK, GFP) was 5′-end labeled with 10 μCi of γ-32P-ATP (3,000 Ci mmol, 10 μCi/μl; PerkinElmer) using T4 PNK.

### Immunoprecipitation and western blotting

For anti-myc immunoprecipitations (IP), we used 5 μg of anti-myc antibody (AM1007a Abgent) coupled with 50 μl of protein G Dynabeads (Thermo) per IP. Antibody-coupled beads were incubated with 293T cells lysates overnight at 4°C. Lysates were prepared from 10^7^ 293T cells, transfected with GARS-myc-expressing constructs, using lysis buffer (50 mM Tris–HCl pH 7.5, 150 mM NaCl, 0.25% NP-40, 2 mM Pefabloc). After IP, the beads were washed three times with the wash buffer (50 mM Tris–HCl pH 7.5, 150 mM NaCl, 0.1% NP-40, 2 mM Pefabloc). 1/20 of the beads were eluted with the SDS-PAGE sample buffer for western blotting analysis with anti-myc antibody, with 3% of inputs and 2.5% of immunoprecipitates loaded on the gel. The rest of the beads were supplemented with 500 ng of GFP spike-in RNA, extracted with Trizol according to the manufacturer's instructions and isolated RNA was analyzed by urea PAGE and northern blotting. For GFP RNA spike-in preparation, a 325-nt fragment of the GFP coding sequence was PCR amplified with the oligos introducing T7 promoter (T7-GFP-fw: TAATACGACTCACTATAGGGATGGTGAGCAAGGGCGAGGA, GFP-rev: GGGTCTTGTAGTTGCCGTCG), and the resulting PCR fragment was used as a template for T7 *in vitro* transcription reaction.

For western blotting, 20 μg of total protein, unless otherwise indicated, was separated on a 4–12% SDS-PAGE, and proteins were transferred to the PVDF membrane. The membrane was probed with the following primary antibodies: rabbit anti-eIF2a antibody 1:1000 (9722 Cell Signaling), rabbit anti-phospho-eIF2a antibody 1:1000 (9721 Cell Signaling), mouse anti-myc 1:5000 (AM1007a Abgent), mouse anti-beta-actin 1:5000 (A2228 Sigma), mouse anti-puromycin 1:4000 (Kerafast 3RH11).

### Bioinformatical data analysis

Analysis of ribosome profiling data was performed using an in-house snakemake based pipeline. First, reads were quality trimmed using trim_galore and filtered for common contaminants (human rRNA sequence (rRNA_U13369.1), tRNA sequences (as predicted by GtRNAdb (27) and selected noncoding RNA sequences from the ENSEMBL ncrna collection). Filtered reads were then analysed using fastqc and mapped to the human genome (GRCh38 version97) using STAR (28). Mapped reads were further analyzed using RiboseQC (https://github.com/ohlerlab/RiboseQC) to obtain P-site cutoffs and counted using a custom htseq-based (29) python script split by annotated gene region, read length and P- and A-site codons. Analysis of codon usage was performed for CDS-mapping reads with 3 nt periodicity (21 nt and 29 nt). For this, counts for each codon in A- or P-site were normalized by the sum of all reads for a given read length, site and sample. Normalized counts were then summed and averaged between different conditions.

## RESULTS

### Defects in protein production are recapitulated by overexpression of CMT-GARS mutants

**Figure 1.**
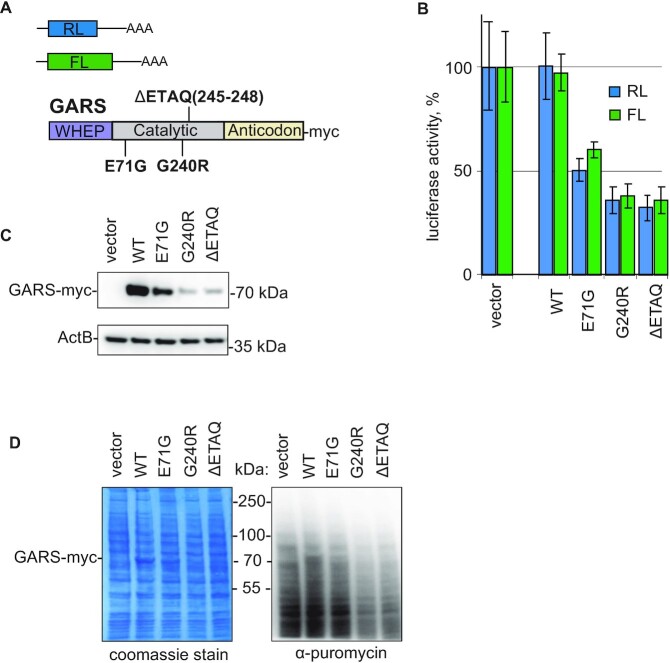
Overexpression of CMT-GARS mutants, E71G, G240R and ΔETAQ, but not WT GARS, represses protein production in cultured cells. (**A**) Schematic representation of constructs used in transfection experiments: RL and FL are reporter constructs encoding *Renilla* and firefly luciferase, correspondingly. Positions of analyzed CMT-GARS mutations in the context of GARS domain structure are shown. (**B**) Repression of RL and FL mRNAs by GARS mutants. Human HEK293T cells were co-transfected with plasmids encoding RL, FL, and myc-tagged GARS, either WT or indicated mutant. As a negative control, empty vector was used instead of GARS-encoding plasmid. RL and FL activities are presented as a percentage of luciferase activity produced in the presence of empty vector. Values represent means ± SD from three experiments. (**C**) Expression levels of myc-fusion proteins were estimated by western blotting with antibodies directed against myc-tag. Beta-actin was used as a loading control. (**D**) Puromycylation assay confirms the role of CMT-GARS in global translational repression. HEK293 cells were transfected with plasmids encoding WT GARS, indicated GARS mutants or an empty vector. After puromycin treatment, cells were lyzed and lysates were analyzed by western blotting with anti-puromycin antibody. PAAG stained with coomassie is provided to visualize equal total protein loading between the samples.

CMT2D is caused by the dominant toxic gain-of-function mutations, i.e. mutations that confer a new and toxic activity on GARS protein (3,5,30). This means that the phenotype of the disease, including defects in translation, can be recapitulated by overexpression of mutant GARS. Thus, we set out to recapitulate the global translational repression triggered by CMT-GARS mutations E71G, G240R (7) and deletion of amino acids 245–248 (ΔETAQ) (31) in cultured HEK293T cells. For that, we co-transfected cells with myc-tagged WT or mutant GARS-encoding plasmids and two reporter constructs coding for *Renilla* and firefly luciferase (RL and FL, Figure 1A). An empty vector was used as a negative control. We found that the overexpression of E71G, G240R and ΔETAQ, but not WT GARS, inhibited protein production in the luciferase reporter assay (Figure 1B). Consistently with their role in global translational downregulation and prior work (7), E71G, G240R and ΔETAQ mutant proteins were expressed at lower levels than WT GARS, as revealed by western blotting (Figure 1C). Interestingly, the reporter assay recapitulated the phenotypic strength of the mutations observed in *in vivo* experiments (7,31): the effects of G240R and ΔETAQ were more severe than that of E71G (Figure 1B and C).

To analyze the effects of CMT-GARS on total translation, we used puromycylation assay, which utilizies puromycin-tagging of newly synthesized proteins (32,33). Puromycin is a mimic of the aa-tRNA, which is incorporated into the nascent polypeptide chains, and the levels of resulting puromycin fusion proteins reflect the rate of translation. To compare translation levels between the cells, transfected with either WT or CMT-GARS mutants, we analyzed cell lysate by western blotting with anti-puromycin antibody (Figure 1D). Indeed, CMT-GARS-expressing cells showed lower incorporation of puromycin (anti-puromycin western), in spite of similar protein loading visualized with coomassie staining. These data confirm that the effect of CMT-GARS mutations on translation is global.

### CMT-GARS mutant inhibits the accommodation of glycyl-tRNA in the ribosomal A-site and causes ribosome stalling

**Figure 2.**
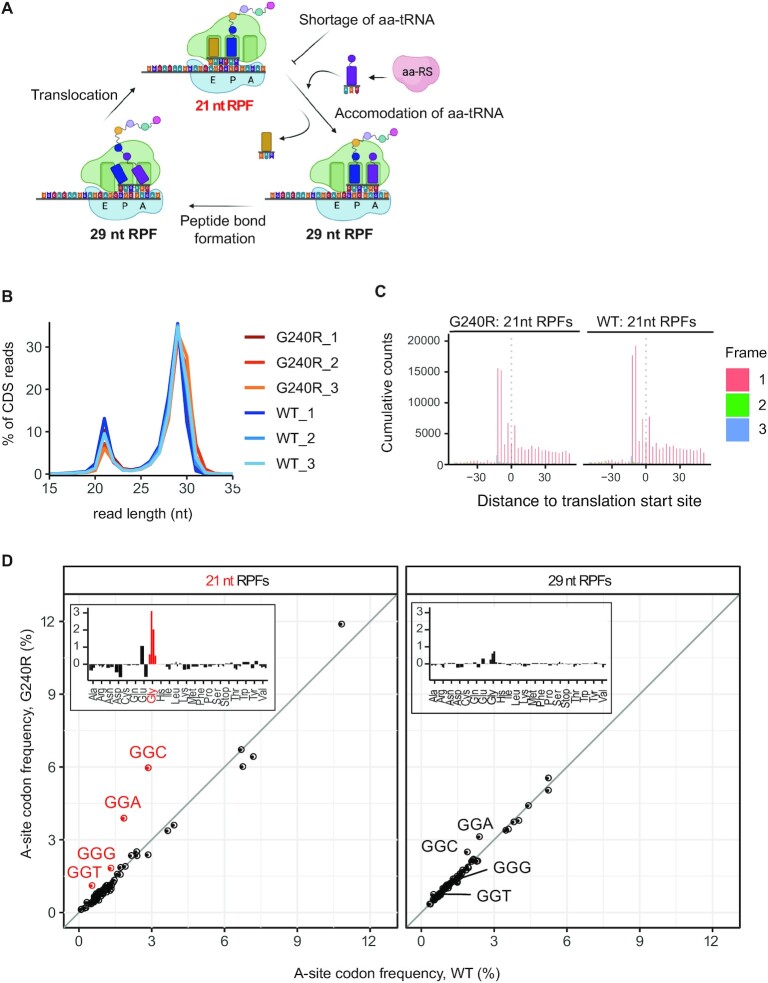
Ribosome profiling detects pausing of ribosome with Gly codons in open A-site in G240R-GARS-expressing cells. (**A**) Scheme showing stages of elongation and the length of ribosome protected fragments (RPFs) generated by each ribosomal state. aa-tRNA: aminoacyl-tRNA, aaRS: aa-tRNA synthetase. (**B**) Length distributions of CDS-mapped ribosome footprints in libraries prepared HEK293T cells expressing either WT or G240R-GARS (in triplicates). (**C**) Metagene aggregate plots displaying distance of 21-nt ribosome footprints from annotated start codon. (**D**) Scatter plots comparing frequencies of 64 codons in the ribosomal A-site between cells expressing WT (X) and G240R-GARS (Y), for 21 nt (left) and 29 nt (right) RPFs. Ribosome frequencies represent the means of triplicates. Glycine codons are labelled and marked in red. Insets show differences between codon frequencies in G240R and WT samples as bar plots.

aa-tRNA synthetases (aaRSs) are required to produce aa-tRNA for the first step of elongation, i.e. accommodation of a cognate aminoacyl-tRNA in the ribosomal A-site (Figure 2A). We decided to test whether this step is affected by CMT-GARS. Our prediction was that, if glycyl-tRNA were deficient in CMT-GARS-expressing cells, ribosomes would stall in a pre-accommodation state once glycine codons entered their A-site. Recent works have shown that high-resolution ribosome profiling can distinguish between different functional states of the ribosome—pre- and post-accommodation of aa-tRNA (20). This technique generates ribosomal footprints on mRNAs; it is achieved by treating cell lysates with RNAse I (34). This degrades most RNA, but leaves ribosome-protected fragments (RPFs) intact; they can then be analyzed by next-generation sequencing. Due to substantial conformational rearrangements of the ribosome during elongation, ribosomes lacking tRNA in their A-sites (open A-sites) generate short 21–22 nt RPFs, while ribosomes with occupied A-sites—produce long 27–29 nt RPFs (20). Thus, the lack of a specific aa-tRNA results in ribosomes with open A-sites pausing on the cognate codons, and this can be detected by high-resolution ribosome profiling by enrichment of the corresponding 21–22 nt RPFs (20) (Figure 2A).

To test this hypothesis, we modified the standard ribosome profiling protocol (34) to isolate a wide range (15–35 nt) of RPFs. Triplicate ribosome profiling libraries were prepared from HEK293T cells expressing a strong CMT-GARS mutant G240R or WT GARS (negative control). Most ribosome profiling reads mapped within coding sequences (CDS), reflecting a fraction of translated mRNAs, and showed high correlation between triplicates (Supplementary Figure S1A and B). We observed a bimodal distribution of ribosome footprints, with the peaks corresponding to the ribosomal states with open (21 nt) and occupied (29 nt) A-sites (Figure 2B). Importantly, both short 21 nt and long 29 nt RPFs showed a periodic alignment pattern of 3 nt, which reflects the codon-by-codon movement of translating ribosomes along an mRNA and represents a hallmark of translation (Figure 2C and Supplementary Figure S1C).

We next calculated the mean frequencies of 64 codons in the A-sites and P-sites of 21 nt and 29 nt RPFs and compared these values between cells expressing WT and G240R GARS (Figure 2D and Supplementary Figure S1D). The most striking difference in codon frequency between G240R and WT GARS samples was detected for 21 nt RPFs with glycine codons in ribosomal A-site (Figure 2D, left). We found that ribosomes paused on glycine codons (red points, Figure 2D) ∼ 2 times longer in G240R than in WT samples, whereas the values for other codons remained similar (black points). Notably, such pausing was observed in short 21 nt RPFs, corresponding to ribosomes with open A-sites, but not in 29 nt RPFs (<1.3-fold, Figure 2D, right), representing ribosomes with occupied A-sites (20). For comparison, only minor changes in codon frequencies were detected in ribosomal P-sites (<1.5-fold, Supplementary Figure S1D). Thus, our ribosome profiling data demonstrate that CMT-GARS mutant G240R induces a stalling of ribosomes with glycine codons in open A-sites, i.e. in a pre-accommodation state. This mechanism is consistent with a shortage of glycyl-tRNA in G240R-expressing cells.

### CMT-GARS mutants have increased capacity to retain bound tRNA^Gly^

**Figure 3.**
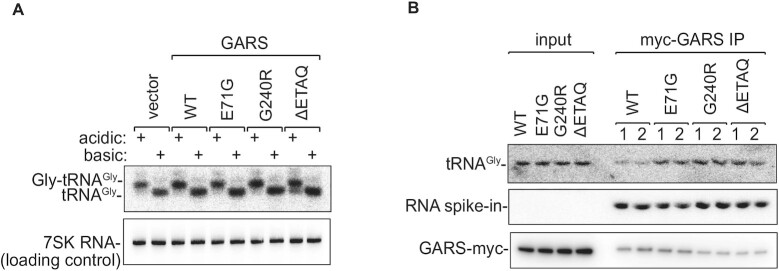
CMT-GARS mutants do not impair overall glycylation activity, but have increased capacity to bind tRNA^Gly^. (**A**) Northern blotting shows that aminoacylation levels of tRNA^Gly^ remain substantially unaffected in the presence of CMT-GARS mutants. HEK293T cells were transfected with plasmids encoding WT, E71G, G240R, ΔETAQ GARS or an empty vector, total RNA was isolated and aminoacylation levels of tRNA^Gly^ were evaluated by acid-urea PAGE and northern blotting. Acidic: lanes showing RNA analysed under acidic pH, that preserves ester bonds linking amino acids to tRNAs. Basic: lanes showing RNA analysed under basic pH, that leads to tRNA deacylation. (**B**) Immunoprecipitation and northern blotting show that CMT-GARS mutants have increased capacity to retain bound tRNA^Gly^. HEK293T cells were transfected with plasmids encoding WT, E71G, G240R or ΔETAQ GARS-myc, with the amounts of plasmid adjusted to achieve equal protein expression. GARS-myc was immunoprecipitated with anti-myc antibody and inputs and immunoprecipitates were analyzed by PAGE and northern blotting to evaluate the levels of GARS-myc-bound tRNA^Gly^. To control for equal efficiency of RNA recovery, immunoprecipitates were supplemented with RNA spike-in before RNA extraction. Duplicates of immunoprecipitates are shown. Western blotting for inputs and immunoprecipitated GARS-myc is shown as a loading control.

Stalling of ribosomes with glycine codons in open A-sites point to a shortage of glycyl-tRNA^Gly^ in G240R-expressing cells. To test if CMT-GARS mutants reduce levels of aminoacylated tRNA^Gly^, we analyzed the levels of Gly-tRNA^Gly^ in CMT-GARS-expressing 293T cells by acid–urea polyacrylamide gel electrophoresis (PAGE) followed by northern blotting. This method allows separation of aminoacylated tRNA from deacylated tRNAs due the mass difference (26). To provide a reference of deacylated tRNA^Gly^, half of each sample was treated with a basic pH buffer, that destabilizes an ester bond between the amino acid carboxyl group and the tRNA terminal 3′-OH group. This analysis showed that most of analyzed tRNA^Gly^ were aminoacylated in 293T cells, and the aminoacylation levels were not substantially altered by expression of either WT or CMT-GARS mutants (Figure 3A). Our results are consistent with the literature data suggesting that CMT-GARS mutations do not disrupt overall aminocylation activity (3–5,7).

Toxic gain-of-function phenotypes can result from increased affinity of the interaction with the natural binders, as observed for example in tauopathies (35). Therefore, we decided to test if a step downstream of aminoacylation, such as the release of tRNA^Gly^ from GARS, is affected by CMT-GARS mutations. To this end, we overexpressed WT GARS and CMT-GARS mutant proteins, tagged with myc-tag, in 293T cells. Given that CMT-GARS mutants are expressed at lower levels than WT (Figure 1C), we adjusted the amounts of transfected plasmids to achieve equal expression of GARS proteins. We followed with immunoprecipitation of GARS-myc fusion proteins with anti-myc antibodies, in duplicates, and analyzed the levels of bound tRNA^Gly^ by PAGE and northern blotting. To control that the efficiency of RNA recovery was the same between the samples, we supplemented the immunoprecipitates with *in vitro* synthesized GFP RNA spike-in before RNA extraction. While efficiency of protein immunoprecipitation (Figure 3B, GARS-myc western) and RNA recovery (RNA spike-in) were similar between the samples, CMT-GARS proteins retained markedly higher amounts of tRNA^Gly^ than WT GARS (tRNA^Gly^). Thus, our data suggest that, due to slow release of tRNA^Gly^, CMT-GARS mutants deplete the pool of glycyl-tRNA^Gly^ available for translation.

### CMT-GARS induces eIF2a phosphorylation and integrated stress response

**Figure 4.**
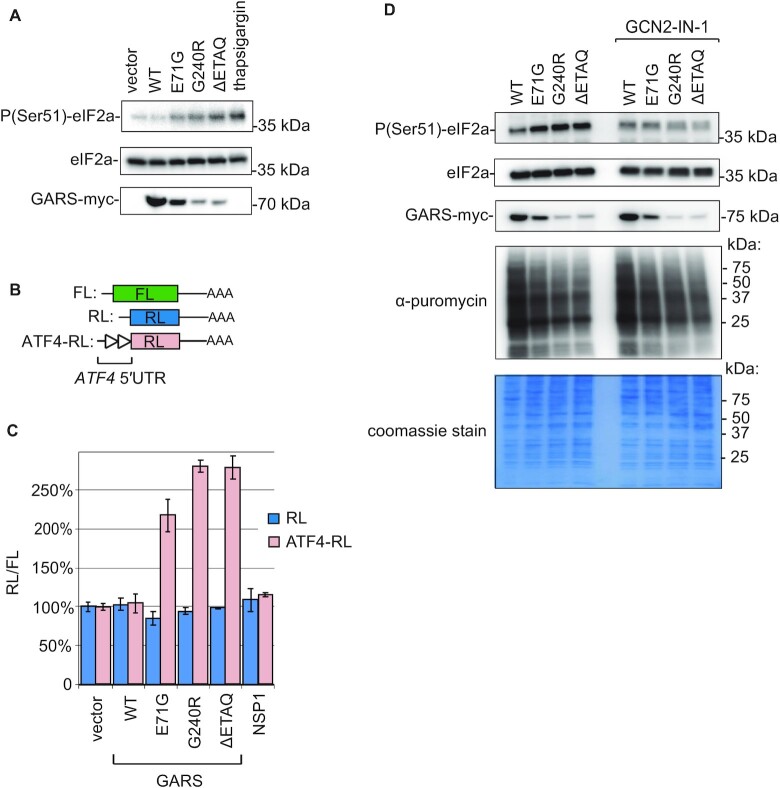
Overexpression of CMT-GARS mutants, E71G, G240R and ΔETAQ, induces integrated stress response (ISR) via phosphorylation of eIF2a. (**A**) Expression of CMT-GARS mutants induces phosphorylation of eIF2a. HEK293T cells were transfected with plasmids encoding WT, E71G, G240R, ΔETAQ GARS or an empty vector. For a positive control of eIF2a phosphorylation, cells were treated with thapsigargin. Cell lysates were analyzed by western blotting using antibodies against phosphorylated eIF2a (P-eIF2a), eIF2a (loading control), and myc (GARS-myc), as indicated on the left. (**B**) Schematic representation of reporter constructs used in transfection experiments: RL and FL are the same as in Figure 2A. ATF4-RL carries 5′UTR of *ATF4* gene. (**C**) E71G, G240R and ΔETAQ GARS mutants activate expression of *ATF4* reporters. Human HEK293T cells were co-transfected with plasmids encoding one of the *Renilla* luciferase reporters (RL, ATF4-RL), FL and myc-tagged GARS, either WT or indicated mutant. As additional controls, empty vector and NSP1-encoding plasmids were used instead of GARS plasmid. RL activity was normalized to that of FL and presented as a percentage of luciferase activity produced in the presence of empty vector for each *Renilla* reporter. Values represent means ± SD from three experiments. (**D**) Puromycylation assay shows that CMT-GARS-mediated translational repression is preserved upon inhibition of eIF2a phosphorylation. HEK293T cells were transfected with plasmids encoding WT, E71G, G240R or ΔETAQ GARS and inhibitor of eIF2a phosphorylation phosphorylation GCN2-IN-1 was added were indicated. After puromycin treatment, cells were lyzed and lysates were analyzed by western blotting with antibodies against phosphorylated eIF2a (P-eIF2a), eIF2a (loading control), myc (GARS-myc) and puromycin, as indicated on the left. PAAG stained with coomassie is provided to show total protein loading.

Amino acid starvation and deacylated tRNAs are known to induce ISR by activating the eIF2a kinase GCN2 (36). Recently, ribosome stalling was reported as an an alternative mechanism that can activate ISR via the CGN2-mediated phosphorylation of eIF2a (15,37). Given our evidence for ribosome stalling in CMT-GARS-expressing cells (Figure 2D), we wondered if this also induced the phosphorylation of eIF2a and ISR. As a test, we used western blotting to analyze the levels of phosphorylated eIF2a (P-eIF2a) in cells expressing either mutant or WT GARS (Figure 4A). For a positive control, we treated HEK293T cells with thapsigargin, a drug that induces the PERK-dependent phosphorylation of eIF2a (38). Indeed, we observed that expression of E71G, G240G and ΔETAQ mutant GARS increased the levels of phosphorylated eIF2a, while the total levels of eIF2a remained unaffected.

eIF2a phosphorylation plays an adaptive role during stress, by shutting down global protein synthesis, to save resources, and by upregulating the translation of specific transcripts, such as *ATF4*, required for stress management (10–12). This upregulation occurs through a mechanism involving upstream ORF (uORFs) in *ATF4* 5′UTR. Under normal conditions these uORFs play an inhibitory role, catching scanning ribosomes before they reach the main ORF. The phosphorylation of eIF2a reduces the amount of ternary complex, required for initiation, and therefore increases the chances of scanning ribosomes to reach the main ORF and initiate translation. To test whether this mechanism is activated by CMT-GARS mutations, we generated *Renilla* luciferase reporter bearing *ATF4* 5′UTR (ATF4-RL) and analyzed how CMT-GARS mutants affect its expression (Figure 4B). *Renilla* reporter without *ATF4* 5′UTR (RL) was used as a negative control, and firefly luciferase reporter (FL) was co-transfected with both *Renilla* reporters as a normalization control. We observed that RL and FL reporters were repressed by E71G, G240R and ΔETAQ, as established earlier (Figure 1B and Supplementary Figure S2). Strikingly, ATF4-RL was resistant to CMT-GARS-mediated repression (Supplementary Figure S2, Figure 4C). Indeed, relative RL/FL expression was upregulated by E71G, G240G and ΔETAQ mutants specifically for ATF4-RL, but not RL that served as a negative control (Figure 4C). As a control translational repressor we used SARS-CoV2 nonstructural protein 1 (NSP1), that inhibits global translation initiation by binding and obstructing the mRNA entry tunnel on the small ribosomal subunit (39,40). Indeed, NSP1 repressed all reporters—ATF4-RL, RL and FL—to a similar extent (Supplementary Figure S2 and Figure 4C), pointing that the upregulation of ATF4-RL is specific to GARS mutants. Thus, our data show that expression of CMT-GARS mutants induces *ATF4* reporter, a marker of ISR.

We next wondered if phosphorylation of eIF2a substantially contributes to translational repression by CMT-GARS mutants. To this end, we analyzed how CMT-GARS proteins affect translation in the presence of the inhibitor of the eIF2a kinase GCN2, GCN2-IN-1 (41). To evaluate translation levels, we used puromycylation assay introduced in Figure 1D. While addition of GCN2-IN-1 indeed suppressed eIF2a phosphorylation (Figure 4D, P(Ser51)-eIF2a), CMT-GARS mutants continued to repress translation under these conditions (anti-puromycin). Our data suggest that the primary defect caused by CMT-GARS, i.e. ribosome pausing on glycine codons, is sufficient to repress global translation.

## DISCUSSION

CMT is the most common inherited neuromuscular disease affecting 1 in 2500 people worldwide (reviewed in (2)). The molecular mechanism of CMT has been obscure. Thus, although CMT-causing heterozygous mutations in the glycyl-tRNA synthetase gene *GARS* affect protein synthesis, loss of aminoacylation activity is neither necessary nor sufficient to cause the disease (3–5,7). Indeed, some of them retain full (E71G) or partial (G240R) aminoacylation activity, and the WT allele of *GARS* produces a fully functional protein (4,5,7). Moreover, the overexpression of CMT-GARS mutants in *Drosophila* caused defects in motor performance, without any reduction in aminoacylation activity and or changes in the ratios between glycylated versus non glycylated tRNAs (7). Additionally, experiments overexpressing WT GARS did not rescue CMT phenotypes in mouse or *Drosophila* models (7,30). These findings suggested that CMT-GARS mutations inhibit translation via some other toxic gain-of-function mechanism which remained enigmatic.

**Figure 5.**
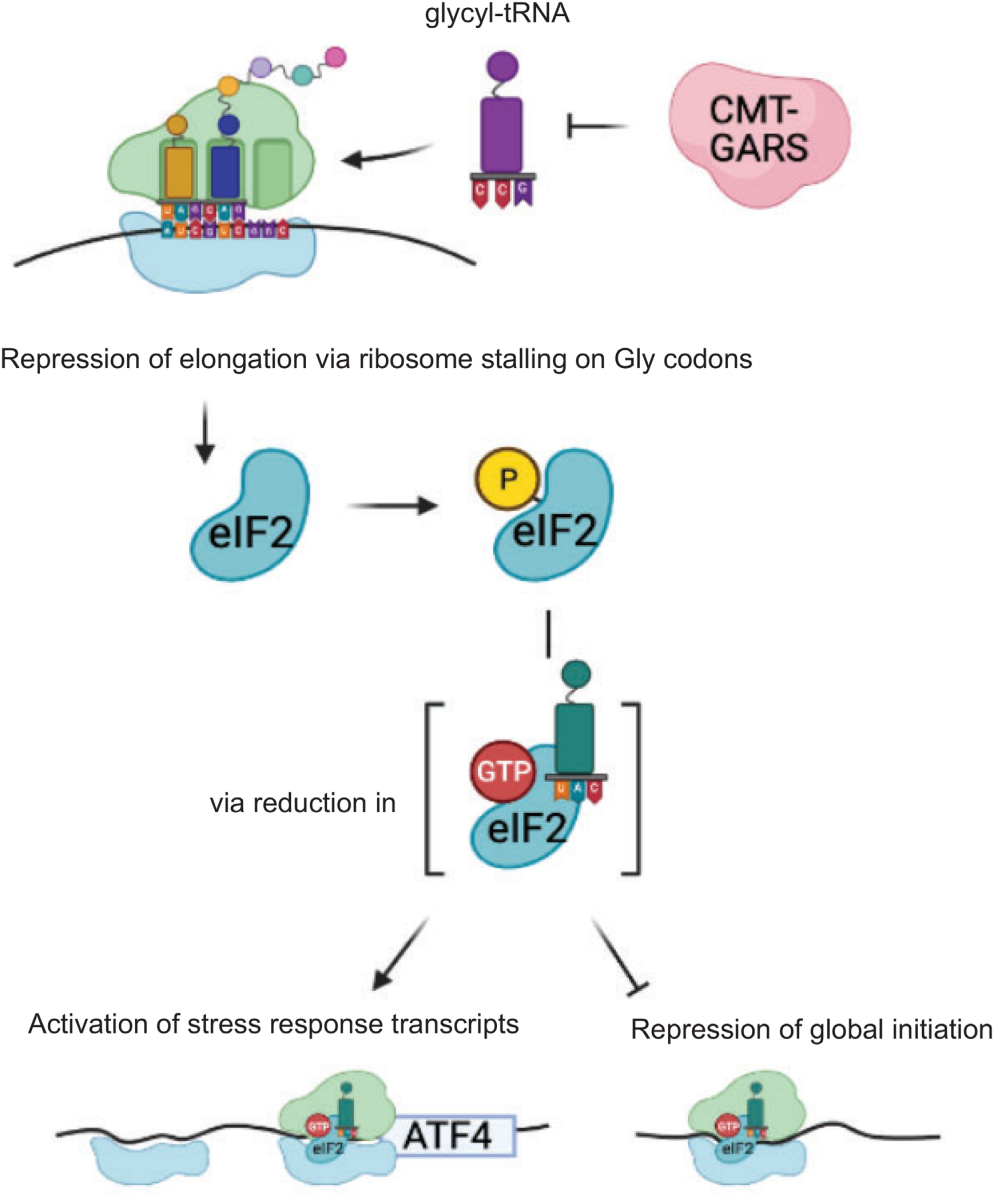
Model illustrating the mechanism of CMT-mutant GARS function in translational regulation. CMT-mutant GARS protein inhibits accommodation of glycyl-tRNA into the ribosomal A-site, possibly via decreasing the pool of available charged glycyl-tRNA, and leads to ribosome stalling on glycine codons. Ribosome stalling results in phosphorylation of eIF2a and activation of integrated stress response. In particular, phosphorylation of eIF2a leads to reduction in the levels of ternary complex eIF2:GTP:Met-tRNAi, which downregulates global translation initiation and upregulates expression of selected transcripts with uORFs, such as *ATF4*. When levels of ternary complex are low, ribosomes bypass uORF, which allows them to initiate translation on the main *ATF4* ORF. ATF4 is a transcription factor that induces stress response genes.

Here, we use high-resolution ribosome profiling to show directly and for the first time that CMT-GARS mutant inhibits the first step of elongation – the accommodation of glycyl-tRNA in the A-site—and thus causes ribosome stalling (Figures 2D and 5). We propose that the degenerative phenotypes observed in CMT can be attributed to this stalling. In other cases, ribosome stalling due to deficiencies in tRNA^Arg^ and ribosome rescue factor GTPBP2 in mouse have been shown to cause neurodegeneration (15).

Our data on ribosome stalling at glycine codons in open A-site point to an insufficiency of glycyl-tRNA^Gly^ in G240R-expressing cells. However, both our results (Figure 3A) and published data (3–5,7) suggest that CMT-GARS mutations do not disrupt aminocylation activity. Toxic gain-of-function mutants can act via different mechanisms, including increased affinity of the interaction with their natural binders, acquiring new abnormal binders or a tendency to aggregate. For example, Alzheimer's disease-associated mutants of a microtubule-binding protein tau bind tubulin heterodimers with enhanced affinity (35). Thus, we considered that a step downstream of aminoacylation, such as the release of glycyl-tRNA^Gly^ from GARS and transfer to eEF1A:GTP for delivery to ribosome, is likely to be affected by CMT-GARS mutations. Indeed, northern blotting of GARS-CMT immunoprecipitates showed that mutant forms of GARS have increased affinity to tRNA^Gly^ (Figure 3B).

While ribosome stalling explains the mechanism of global translational repression by CMT-GARS, we show that it also activates a secondary mechanism of repression at the level of initiation, by inducing ISR via eIF2a phosphorylation (Figure 4A). Reports have shown that stalled ribosomes are more potent activators of the eIF2a kinase GCN2 than deacylated tRNAs, which result from amino acid starvation (15,37). Interaction with ribosomal P-stalk of stalled ribosomes is suggested to activate GCN2 (37), although the exact mechanism of discrimination between translating and stalled ribosomes remains unclear. The phosphorylation of eIF2a prevents the formation of the ternary complex and thus inhibits global translation initiation, to save cellular resources (Figure 5). Beyond that, it enhances the translation of specific mRNAs, such as *ATF4*, which contains uORFs in it 5′UTRs (10–12). ATF4 promotes the transcription of genes with adaptive functions that can repair damage caused by stress (42,43). However, a chronic activation of ISR can contribute to neurodegenerative phenotypes through the induction of apoptosis, memory impairments due to translational inhibition and other mechanisms (reviewed in (1)). For example, *ATF4* deficiency was shown to alleviate neuronal loss from oxidative stress and amyloid beta peptide (44,45).

It remains unclear why mutations in ubiquitously expressed aaRSs primarily affect peripheral motor and sensory axons. The fact that the expression of CMT-GARS mutants in a heterologous system permits recapitulating the repression of protein production, characteristic of CMT (Figure 1B), suggests that the mechanism *per se* is not unique to a single cell type. One possible explanation for the higher susceptibility of peripheral motor and sensory axons might be that they have low amounts of some of translation components that are involved in this mechanism (e.g. tRNAs, aaRSs etc.). Further studies aimed at identifying such limiting components are likely to elucidate the cell specificity of this mechanism.

There is no cure for CMT and understanding of the mechanisms of the CMT-GARS function opens new perspectives for development of therapies. Antisense oligos (ASO) are a promising therapeutic strategy to downregulate genes with toxic gain-of-function phenotype (reviewed in (46)). ASO injections produced encouraging results in treatment of several neurological diseases, including spinal muscular atrophy and Huntington's disease. In case of CMT however, this approach would require generation of individualized ASOs recognizing specific CMT-GARS mutations, which in most cases differ from a WT allele in a single nucleotide, making the ASO design complicated. An alternative approach to therapies is targeting different steps of the CMT-GARS-mediated translational repression. Our data on ribosome stalling at glycine codons in open A-site point to the shortage of glycyl-tRNA as a mechanism of CMT. Therefore, providing a supply tRNA^Gly^ could be a therapeutic strategy to alleviate ribosome pausing. Activation of ISR may also contribute to neurodegenerative phenotypes in CMT, supported by data from other neurodegenerative diseases (reviewed in (1)). Indeed, drugs targeting ISR and alleviating translational repression have been shown to efficiently reduce neurodegeneration symptoms in various models (13) and may be a promising approach for treatment of CMT.

## DATA AVAILABILITY

RiboseQC tool used for data analysis is available at the github: https://github.com/ohlerlab/RiboseQC. Ribosome profiling data are deposited at the ArrayExpress (accession E-MTAB-10342).

## Supplementary Material

gkab730_Supplemental_File
