# Supplementary material for: Charcot–Marie–Tooth mutation in glycyl-tRNA synthetase stalls ribosomes in a pre-accommodation state and activates integrated stress response
Source: Nucleic Acids Res. 2021 Aug 17;49(17):10007–17. doi: 10.1093/nar/gkab730 (PMC8464049; doi:10.1093/nar/gkab730)
Supplement: gkab730_Supplemental_File [file gkab730_supplemental_file.pdf]

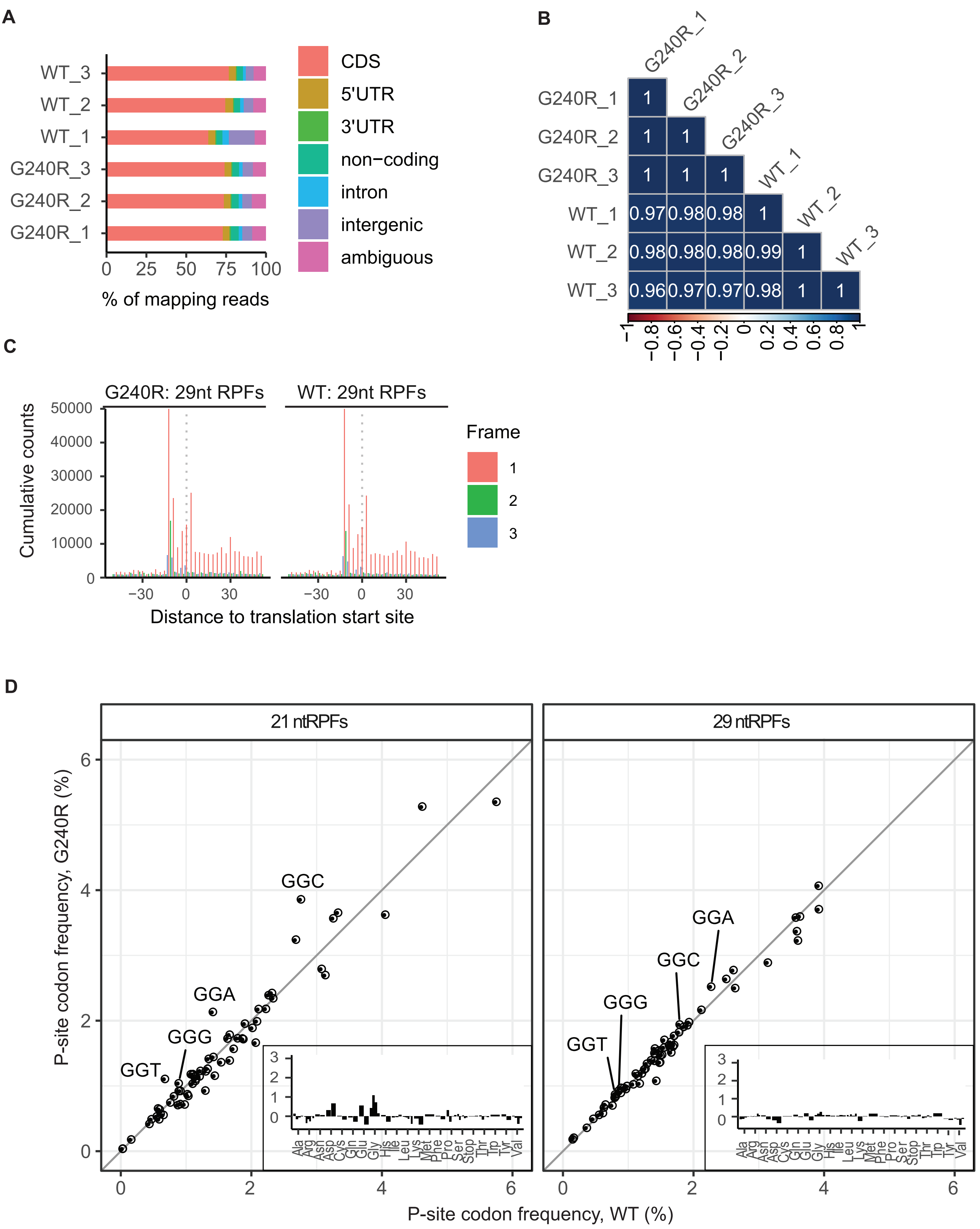

Figure S1. Ribosome profiling of HEK293T cells expressing G240R mutant and WT GARS. (**A**) Bar plot depicting mapping statistics of ribosome profling reads. Most reads map to CDS, as expected from reads derived from translated mRNAs. (**B**) Correlation heatmap of individual ribosome profiling libraries: high correlation is observed between the replicates. The numbers represent Pearson correlation coefficients (reads/gene in CDS). (**C**) Metagene plots showing the percentage of 29-nt ribosome footprints from annotated start codon. (**D**) Scatter plots comparing frequencies of 64 codons in the ribosomal P-site between cells expressing WT (X) and G240R-GARS (Y), for 21 nt (left) and 29 nt (right) RPFs. Ribosome frequencies represent the means of triplicates. Glycine codons are labelled. Insets show differences between codon frequencies in G240R and WT samples as bar plots.

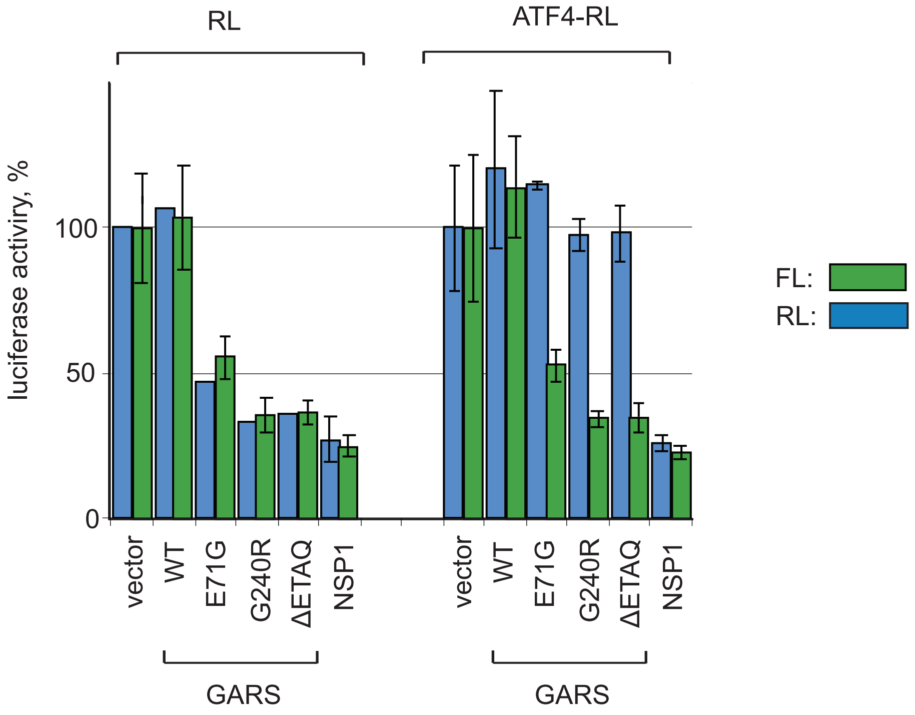

Figure S2. Overexpression of CMT-GARS mutants upregulates ATF4 reporter. The experiment was performed as described in **Figure 3C**, but data are shown separately for RL and FL reporters. In short, HEK293T cells were co-transfected with plasmids encoding one of the Renilla luciferase reporters (blue: RL, ATF4-RL), FL (green), and myc-tagged GARS, either WT or indicated mutant. As additional controls, empty vector and NSP1-encoding plasmids were used instead of GARS plasmid. RL and FL activities are presented as a percentage of luciferase activity produced in the presence of an empty vector. Values represent means +/- SD from 3 experiments.
